# Application of Plaster-of-Paris Bandages

**Published:** 1880-02

**Authors:** 


Application of Plaster-of-Paris Bandages.—The following mode of applications of plaster-of-Paris bandages for the treatment of spinal disease, I have found very successful when the displacement is in the lower dorsal or lumbar region. The patient, stripped to about two or three inches below the hips, is placed under the head; two jack or round towels are passed under the armpits, and fastened to the opposite legs of the table to the arms under which they pass, so as to cross over the chest. The head end of the table is then raised until the plane of the table makes an angle of 45° with the floor, and is retained in that position by putting a Windsor chair under each leg. In a few minutes, the spine straightens out, and any displacement of the vertebræ is reduced, when the bandages can be applied without hurry to the attendants or inconvenience to the patient. The table should be covered with a blanket.—*British Medical Journal.*

